# An unusual case of Ebstein anomaly in 72 year old woman

**DOI:** 10.1186/1749-8090-8-S1-P97

**Published:** 2013-09-11

**Authors:** E Ntalarizou, K Dimopoulos, I Saeed, L Swan, H Uemura

**Affiliations:** 1 Congenital Heart Disease Surgery, Royal Brompton and Harefield NHS Foundation Trust, London, UK; 2 Cardiology Department, Royal Brompton and Harefield NHS Foundation Trust, London, UK; 3 Adult Congenital Heart Disease Cardiology Department, Royal Brompton and Harefield NHS Foundation Trust, London, UK

## Background

Ebstein’s anomaly is a rare congenital heart disorder (1 per 200,000 live births), accounting for about 0.3 to 0.7% of all cases of congenital heart disease. The condition is characterized by tricuspid valve abnormality with apical displacement of the septal and posterior leaflets from the atrioventricular annulus into the right ventricle, leading to the ‘atrialization’ of a portion of the right ventricle. Only 5% of patients with Ebstein’s anomaly survive beyond the fifth decade while original presentation in older patients is rare.

## Methods

Single case report of a 72-year-old woman with Ebstein's anomaly and concomitant ASD. Surgical management is discussed.

## Results

A 72 year old Caucasian female presented to our centre with palpitations and shortness of breath in the context of a one year history of fatigue and exhaustion. She underwent atrial flutter ablation and a permanent dual chamber pacemaker implantation 3 years prior to her admission and diagnosed with Ebstein malformation and ASD. Chest radiograph revealed cardiomegaly with a CTR of 17.9/28.9 and prominent pulmonary markings. Computed tomography thorax revealed marked right atrial dilatation and abnormal position of the septal leaflet of the tricuspid valve. On echocardiography the left ventricle was small (LVEDD 42mm) with normal systolic function while the septal leaflet of the tricuspid valve was displaced by 26mm (14mm/m^2^) consistent with Ebstein's anomaly. There was moderate-severe tricuspid regurgitation. She underwent surgical management in the form of a tricuspid valve replacement with a bioprosthetic valve and plication of the atrialized ventricle, without aortic cross-clamp. The patient had an uneventful postoperative recovery.

## Conclusion

Our case highlights that Ebstein’s anomaly can present later in life; regular long-term cardiac follow up with an experienced cardiologist is required to prevent further cardiac complications.

